# Establishment and Maintenance of Conventional and Circulation-Driven Lung-Resident Memory CD8^+^ T Cells Following Respiratory Virus Infections

**DOI:** 10.3389/fimmu.2019.00733

**Published:** 2019-04-05

**Authors:** Shiki Takamura, Jacob E. Kohlmeier

**Affiliations:** ^1^Department of Immunology, Faculty of Medicine, Kindai University, Osaka, Japan; ^2^Department of Microbiology and Immunology, Emory University School of Medicine, Atlanta, GA, United States

**Keywords:** tissue-resident memory, CD8^+^ T cells, memory T cell maintenance, lung, respiratory virus infections

## Abstract

Antigen-specific CD8^+^ tissue-resident memory T cells (T_RM_ cells) persist in the lung following resolution of a respiratory virus infection and provide first-line defense against reinfection. In contrast to other memory T cell populations, such as central memory T cells that circulate between lymph and blood, and effector memory T cells (T_EM_ cells) that circulate between blood and peripheral tissues, T_RM_ cells are best defined by their permanent residency in the tissues and their independence from circulatory T cell populations. Consistent with this, we recently demonstrated that CD8^+^ T_RM_ cells primarily reside within specific niches in the lung (Repair-Associated Memory Depots; RAMD) that normally exclude CD8^+^ T_EM_ cells. However, it has also been reported that circulating CD8^+^ T_EM_ cells continuously convert into CD8^+^ T_RM_ cells in the lung interstitium, helping to sustain T_RM_ numbers. The relative contributions of these two mechanisms of CD8^+^ T_RM_ cells maintenance in the lung has been the source of vigorous debate. Here we propose a model in which the majority of CD8^+^ T_RM_ cells are maintained within RAMD (conventional T_RM_) whereas a small fraction of T_RM_ are derived from circulating CD8^+^ T_EM_ cells and maintained in the interstitium. The numbers of both types of T_RM_ cells wane over time due to declines in both RAMD availability and the overall number of T_EM_ in the circulation. This model is consistent with most published reports and has important implications for the development of vaccines designed to elicit protective T cell memory in the lung.

## Introduction

Memory CD8^+^ T cells in non-lymphoid tissues are optimally positioned to mediate rapid responses to invading pathogens. They comprise at least two distinct subpopulations: tissue-resident memory T cells (T_RM_ cells) and effector memory T cells (T_EM_ cells). T_RM_ cells are a non-circulating population that typically, but not exclusively, expresses a specific array of surface markers (e.g., CD69, CD103, and CD49a) and possess gene-expression profiles that are associated with tissue retention (1). In contrast, T_EM_ cells lack the expression of these molecules and continuously circulate between blood and non-lymphoid tissues (2). The vast majority of memory CD8^+^ T cells in most non-lymphoid tissues are T_RM_ and play the predominant role in protective immunity (3, 4). In contrast, memory CD8^+^ T cells in the circulation have minimal, if any, impact on immediate local protection (3, 5). However, it is possible that the small numbers of CD8^+^ T_EM_ cells that transit through the tissues at the time of reinfection may contribute to protection.

The lung appears to differ from other non-lymphoid tissues in that it harbors relatively large numbers of both tissue-circulating T_EM_ and T_RM_ cells in a number of distinct niches (3, 6). Furthermore, these memory CD8^+^ T cell subpopulations alter their phenotypes and functions in response to environmental factors present in distinct compartments of the lung (7, 8). Thus, a complete understanding of the phenotypic and functional features of these memory T cell populations in each of these lung compartment has been hampered by the challenges of isolating pure populations for analysis. This has resulted in confusion in the field. In this perspective, we attempt to resolve these issues and outline a model that explains the generation and maintenance of diverse populations of memory CD8^+^ T cells in the lung.

## Memory CD8^+^ T Cells in the Lung

The tissues that comprise the barrier surfaces of the body typically consist of an epithelial layer that overlays a stromal layer, such as the epidermis and dermis in the skin and the epithelium and lamina propria in the intestine. These tissues differ considerably and provide distinct anatomical and biological niches for the maintenance of memory CD8^+^ T cells (9). Consistent with other barrier tissues, the lung airways (epithelium) and the lung interstitium (stroma) host phenotypically and functionally distinct memory CD8^+^ T cell populations.

Memory CD8^+^ T cells in the lung airways are localized primarily in the epithelial layers of the bronchiole and are readily isolated by bronchoalveolar lavage (BAL) (10–12). Since the lung airways are anatomically separated from blood vessels, there are few, if any, blood cell contaminants in BAL samples (unless the blood vessels are damaged by poor technique or infection). Consequently, it is possible to interpret the data on T cells isolated by BAL without using intravascular (i.v.) staining to distinguish contaminating cells from the blood (13). Such BAL data indicate that large numbers of antigen-specific memory CD8^+^ T cells are present in the lung airways for several months following recovery from a respiratory virus infection (14, 15). These airway T cells do not return to the circulation or the lung interstitium under steady-state conditions (12), suggesting that they are T_RM_. However, since they have relatively short lifespans (presumably due to cell-extrinsic factors, such as their biophysical removal by the barrier function of airway mucosa) (16), their maintenance depends on continual influx of memory cells from the interstitium (16, 17). This continual replenishment of memory pool does not fit with the definition of T_RM_ and, as such, is a unique feature memory CD8^+^ T cells in the lung airways. Upon recruitment to the airways, the cells receive antigen-independent local environmental cues to acquire an activation phenotype (e.g., upregulation of CD69) and to completely downregulate the integrin LFA-1 (CD11a) (7, 16). As a result, memory CD8^+^ T cells in the airways lose cell contact-mediated cytolytic activity (11). Nevertheless, these cells can confer antigen-specific protection by rapidly secreting interferon (IFN)-γ in the face of antigenic challenge (18, 19).

**Figure 1:**
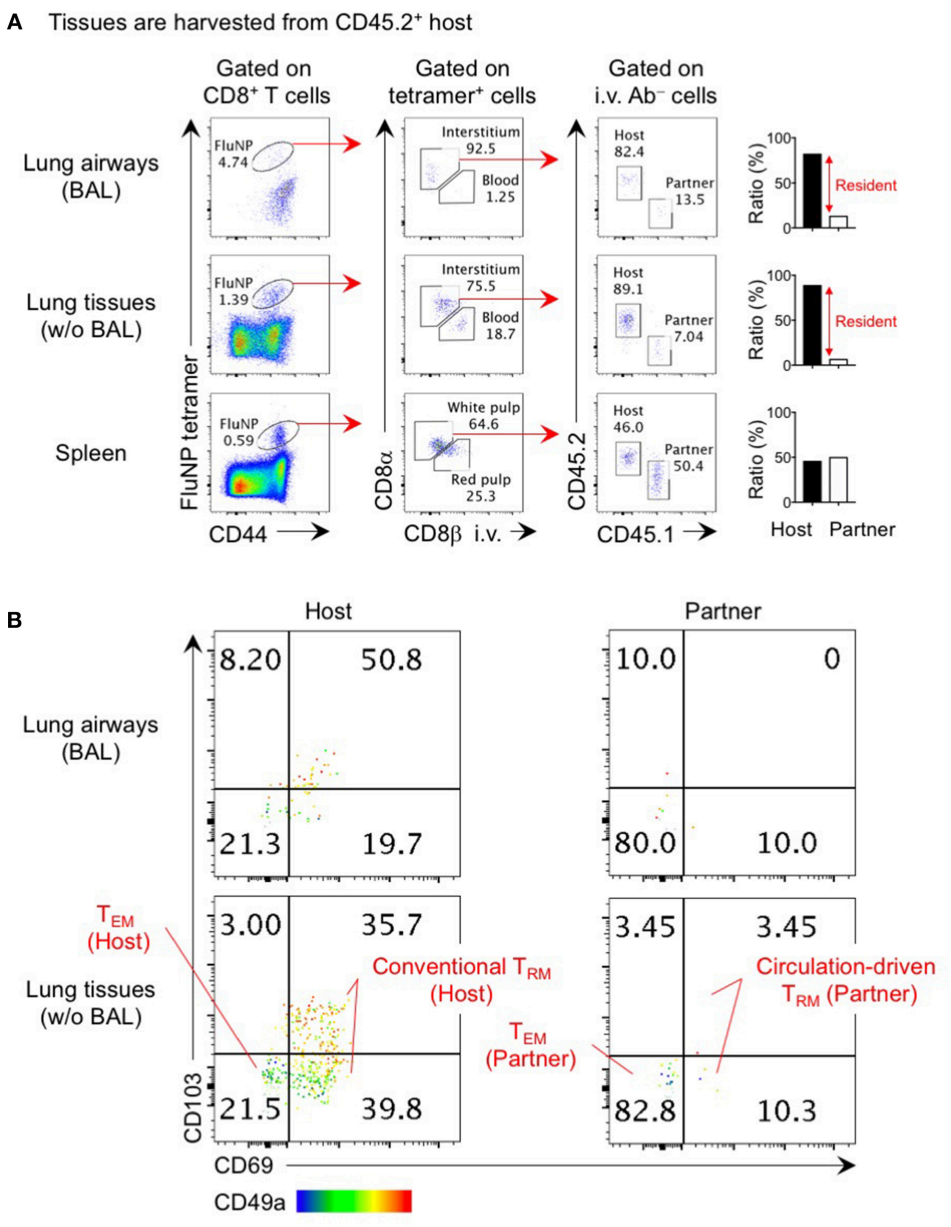
Analysis of lung T_RM_ and T_EM_ by parabiosis experiment. Congenically distinct mice (CD45.1^+^ and CD45.2^+^) were infected i.n. with influenza virus x31 (300 EID_50_) and subjected to parabiotic surgery 35 days later. Day 21 after the surgery, mice were injected i.v. with 1 μg anti-CD8β 3 min prior to tissue harvest. Cells in the lung airways were recovered by BAL. Lung tissues were digested by collagenase D, and enriched by centrifugation in 40/80% Percoll gradient. Cells were stained with influenza NP_366−374_/D^b^ tetramer and fluorescent-conjugated antibodies. Data shown are derived from a CD45.2^+^ parabiont. Plots shown in **(A)** indicate the gating strategy of host- and partner-derived antigen-specific CD8^+^ T cells in the spleen, lung interstitium and airways. Bar graphs show ratio of host and partner cells among i.v. antibody negative cells in individual mouse. Plots shown in **(B)** indicate the expression of CD69, CD103, and CD49a on host- and partner-derived NP_366−374_/D^b^ tetramer^+^ CD8^+^ T cells in the lung airways and interstitium. Host cells are the mixture of a large proportion of T_RM_ (CD69^+^ CD49a^+^ CD103^+^ and CD69^+^ CD49a^+^ CD103^−^) and a minor population of T_EM_ (CD69^−^ CD49a^−^ CD103^−^). The former population may include a small number of circulation-driven T_RM_ converted from host T_EM_. The data also show how circulation-driven T_RM_ cells are a relatively small population and are difficult to identify in individual animals.

Memory CD8^+^ T cells in the lung interstitium can be purified by enzymatic digestion of lung tissues after removal of the BAL. However, cells prepared this way are contaminated with small numbers of memory CD8^+^ T cells that had been trapped in the airways, although a certain fraction of these cells (i.e., cells that are exposed in the airway environment more than 48 h) can be distinguished by their reduced expression of CD11a (16). Interstitial cells prepared by enzymatic digestion are also contaminated with blood derived T cells from the capillaries. Therefore, prior i.v. staining is necessary to discriminate cells in the interstitium from those in the pulmonary capillary bed (13). It is important to point out two things here. First, data regarding parenchymal cells that have been isolated without i.v. staining must be cautiously interpreted given the significant degree of blood cell contamination. For example, before researchers began discriminating cells in the lung tissue and the lung vasculature, lung interstitium had erroneously been considered to be a “permissive tissue” that was readily accessible to memory CD8^+^ T cells in the circulation (20–22). However, a more detailed analysis has revealed that, as with other mucosal tissues, the migration of circulating memory CD8^+^ T cells into the lung interstitium is minimal in uninfected lung interstitium (6, 23, 24). Second, because memory CD8^+^ T cells in the lung interstitium (i.e., negative for i.v.-injected antibody) include both T_RM_ and small numbers of tissue-circulating T_EM_, parabiosis approaches are necessary to distinguish these populations. Using these approaches, we and others have formally demonstrated that a large proportion of memory CD8^+^ T cells in the lung interstitium are T_RM_ cells (Figure 1) (3, 6). It has also become evident that CD8^+^ T_RM_ and T_EM_ cell populations are maintained in distinct compartments of the lung interstitium: the former is predominantly localized within the site of tissue repair and regeneration around the bronchiole (we termed these Repair-Associated Memory Depots: RAMD), while the latter are widely and sparsely distributed in unaffected areas of the interstitium (6). Unlike memory CD8^+^ T cells in the airways, CD8^+^ T_RM_ cells in the lung interstitium are a stable population (6). Hence, memory CD8^+^ T cells in the lung interstitium comprise a mixture of stable (T_RM_) and dynamic (T_EM_) memory populations that are maintained independently.

The true phenotypes of memory T cells in the lung interstitium are best revealed through parabiosis studies in which a pair of influenza virus infected mice are surgically joined after memory has been established and rested until leucocytes in the blood of each mouse are equilibrated. Non-circulating host CD8^+^ T cells in the lung predominantly, but not exclusively, express T_RM_ markers CD69, CD103, and CD49a that facilitate tissue-retention while partner-derived T_EM_ cells are mostly negative for these markers (Figure 1) (6). The small fraction of host CD69^−^ CD103^−^ CD49a^−^ cells likely represent the host-derived T_EM_ population. It is interesting that a sizable fraction of host CD69^+^ CD49a^+^ cells in both the lung interstitium and airways lack the expression of another T_RM_ marker, CD103 (Figure 1) (6, 25). The lack of CD103 on some T_RM_ is consistent with subpopulation of T_RM_ found in the intestinal lamina propria, brain, and liver (26–28). In this regard, i.n. infection of CD103 knockout mice with influenza virus resulted in partial, but not complete, loss of CD8^+^ T_RM_ in these tissues (29). These data also indicate that the CD103 marker does not efficiently discriminate T_RM_ from T_EM_ in the lung. Given the diversity of memory CD8^+^ T cell populations in the lung, it is critical to precisely identify each population to avoid misinterpretation and confusion.

## Generation and Maintenance of Canonical T_RM_ Cells in the Lung

Following initial priming in the draining lymph nodes (LN), effector CD8^+^ T cells migrate to the inflamed tissues where they receive local instructive signals that promote their subsequent differentiation into T_RM_ (9, 30). Transforming growth factor-β1 (TGF-β) is a common factor in most non-lymphoid tissues that drives T cell expression of CD103 and thereby promotes integrin αEβ7-mediated adhesion to E-cadherin on epithelial cells. A variety of cells, such as macrophages and stromal cells in the interstitium, and epithelial cells, are known to produce the latent form of TGF-β in the lung during early phases of an influenza virus infection (25, 31). As with the intestine (32–34), CD103^+^ dendritic cells (DC) in the lung interstitium may play a role in the local conversion of TGF-β into the active form through integrin αvβ8, and promote the establishment of CD103^+^ CD8^+^ T_RM_ cells in the lung (35). In the absence of TGF-β signaling, CD8^+^ T_RM_ cells in the whole lung (i.e., a mixture of cells in the airway and interstitium) completely lack the expression of CD103 (35, 36), although the number of antigen-specific CD8^+^ T cells in the whole lung is not affected (36). This suggests that the establishment of CD103^−^ CD8^+^ T_RM_ cells in the lung interstitium and airways is not dependent of TGF-β.

Since there is limited space for cells to inhabit in normal lung tissue, newly created anatomical niches are required for the establishment and long-term maintenance of CD8^+^ T_RM_ cells in the lung (6, 9, 37). Upon respiratory virus infection, infection-induced cytolysis and disruption of infected cells by antigen-specific effector CD8^+^ T cells both contribute to tissue damage. A broad spectrum of cells including immune cells as well as basal cells (e.g., distal airway stem cells) accumulate at sites of damage to mediate the repair process which can be virtually observed as cytokeratin-expressing cell aggregates (Krt-pods) (38), thereby providing special niches for the establishment of CD8^+^ T_RM_ cells in the lung interstitium (6, 37). Thus, lung CD8^+^ T_RM_ cells may be specifically committed to protect weak spots (tissues undergoing repair) in the lung against reinfection (24). The structural characteristics of these T_RM_ depots (RAMD) differ from inducible bronchus-associated lymphoid tissue (iBALT) as most CD8^+^ T_RM_ cells in the RAMD do not form organized lymphoid structures (iBALT consists of CD4^+^ T cell cluster that surround B cell follicles) (6). This is consistent with the fact that unlike CD4^+^ T cells and B cells that act cooperatively, CD8^+^ T_RM_ cells can act alone upon recall. Furthermore, our timed parabiosis approach (joining pairs of mice at various time points before and after infection) clearly demonstrated that CD8^+^ T cells recruited to the lung later than the peak of T cell response in the lung (around day 10 post influenza virus infection) failed to from T_RM_ (6). This indicates that lung T_RM_ niches are occupied at the peak of tissue damage and are no longer available for latecomer CD8^+^ T cells including T_EM_ cells. It is well known that CD8^+^ T_RM_ cells in the lung display relatively shorter longevity relative to T_RM_ in other tissues as T_RM_ cell-mediated heterosubtypic immunity to influenza virus lost at 4–6 months post-infection (5, 8). The decline in the size of the RAMDs overtime as tissue repair proceeds would explain the limited longevity of lung CD8^+^ T_RM_ cells as compared to CD8^+^ T_RM_ cells in other non-lymphoid tissues (6, 37). Similarly, the elevated proapoptotic activities of CD8^+^ T_RM_ cells in the whole lung can be attributed to the concomitant loss of environmental factors that potentially support the homeostasis of T_RM_ (8).

It has been established that concurrent CD4^+^ T cell responses also contribute to the establishment of CD8^+^ T_RM_ cells in the lung (39). In contrast to other mucosa (female reproductive tract) where CD4^+^ T cells play an indirect role in promoting optimal positioning of CD8^+^ T_RM_ cells by triggering the local production of inflammatory chemokines (40), CD4^+^ T cell help in the lung confers prolonged survival and improved functionality of CD8^+^ T cells by transcriptionally modulating the metabolism to maintain higher spare respiratory capacity (41), a hallmark of T cell memory (42). CD4^+^ T cell-derived IFN-γ also acts directly on CD8^+^ T cells to downregulate the expression of T-bet. This leads to memory CD8^+^ T cell rescue from T-bet-mediated repression of CD103, thereby promoting T_RM_ formation (43). Given the differential distribution of CD8^+^ and CD4^+^ T_RM_ (RAMD and iBALT, respectively), it seems likely that the primary involvement of CD4^+^ T cell help during CD8^+^ T_RM_ formation is exerted during the acute phase of infection (41).

A recent study has shown that cell-intrinsic factors also contribute to the durability of T_RM_ in the lung. CD8^+^ T_RM_ cells generated from memory CD8^+^ T cells that had previously experienced multiple antigen encounters exhibit superior longevity compared to these generated from naïve CD8^+^ T cells (44). Reciprocal adoptive transfer approaches using a mixture of memory and naïve T cell receptor (TCR) transgenic T cells revealed that T_RM_ cells derived from memory cells preferentially occupy lung T_RM_ niches compared to T_RM_ cells derived from naïve cells (44). This suggests that there may be increased frequencies of T_RM_ precursors (KLRG1^lo^ effector cells) among memory-derived CD8^+^ T cells, compared to naïve CD8^+^ T cells following activation in the draining LN. It is also possible that memory-derived CD8^+^ T cells may be capable of receiving additional instructive signals, such as 4-1BB signals for up-regulation of pro-survival factors, when cells are recruited to the RAMD and acquire resultant longevity (45).

Cognate antigen-driven local reactivation is also indispensable for the establishment of lung CD8^+^ T_RM_ cells. The best example for this is the impact of route of infection/vaccination on the establishment of CD8^+^ T_RM_ cells in the lung. Intranasal (i.n.) infection elicits robust populations of CD8^+^ T_RM_ cells in the lung interstitium and airways, whereas non-pulmonary route of infection do not (5, 6, 19, 23, 24, 35, 46–49). In the case of the skin and genital tract, forced recruitment of circulating CD8^+^ T cells to the mucosa using inflammatory stimuli or topical administration of chemokines is sufficient to establish local T_RM_, an approach referred to as “prime and pull” (50, 51). However, we and others have shown that the exposure of CD8^+^ T cells to the lung environment is insufficient to promote subsequent differentiation of these into long-lived lung T_RM_ (6, 23, 35). Instead, local reactivation induced by pulmonary administration of trace amount of antigen during the process of “prime and pull” is necessary for converting circulating CD8^+^ T cells into lung T_RM_ cells (6, 23). Thus, both cell-intrinsic and extrinsic factors are necessary for complete conversion of these cells to T_RM_. First, pulmonary administration of antigen generates antigen-bearing target cells that are eliminated by antigen-specific CD8^+^ T cells, leading to the creation of damage and repair-associated T_RM_ niches (6). Second, local reactivation provides cell-intrinsic effects such as prolonged expression of CD69 and CD49a necessary for retention (6, 23), and upregulation of interferon-induced transmembrane protein 3 (IFITM3) for survival (52). Furthermore, TCR signaling may protect T_RM_ cells from a damage/danger-associated molecular pattern (DAMP)-induced cell death (53). Interestingly, there is differential expression of CD103 on distinct epitope-specific CD8^+^ T_RM_ cells in the lung, irrespective of their localization, suggesting that difference in the extent of antigen presentation or subset of antigen presenting cells (APC) involved may also influence lung T_RM_ biology (25).

While it is unclear which APC provide local antigen signaling, the delivery of antigen to pulmonary DC by antibody-targeted vaccination (conjugate of antigen and antibody specific for DC) significantly facilitates the establishment of CD103^+^ CD8^+^ T_RM_ cells in the lung (35). Furthermore, CD103^+^ respiratory DC are known to continually carry residual antigen from the lung to the draining LN, suggesting that respiratory DC are the primary source of local antigen signaling (54). Given the unique ability of CD103^+^ respiratory DC to provide strong stimulatory signals in the draining LN, thereby generating effector CD8^+^ T cells that preferentially home back to the lung (55), local reactivation by respiratory DC may promote terminal effector maturation rather than memory differentiation (56–58). Thus, other APC subsets, such as pulmonary macrophages, that accumulate in the RAMD during the early phase of infection may be necessary to provide the optimal antigen signaling required for T_RM_ development (59, 60).

## Conversion From T_EM_ to T_RM_: A Minor Pathway of T_RM_ Development in the Lung

Despite the inefficiency of the non-pulmonary route of infection/immunization in establishing lung CD8^+^ T_RM_ cells, several studies have nevertheless reported the deposition of CD8^+^ T_RM_ cells in the lung following systemic infections (3, 61–63). Such blood-borne T_RM_ are derived from effector cells that have undergone less differentiation (defined as null to intermediate expression of CX3CR1 and lack of KLRG1 expression and including exKLRG1 cells that have downregulated this molecule during the contraction phase) (64, 65). Adoptive transfer of splenic memory clearly revealed the emergence of a small fraction of CD103^+^ CD69^+^ CD8^+^ T cells in the whole lung (8). The appearance of CD8^+^ T cells exhibiting T_RM_ phenotypes was also evident in our parabiosis experiments (Figure 1) (6), indicating that some levels of T_EM_ to T_RM_ conversion occurs in the lung. These cells exhibited a T_RM_ gene-expression signature and their tissue-residency was also confirmed by parabiosis (3, 61). Since several cytokines, such as TGF-β, IL-33, and tumor necrosis factor (TNF)-α, are reported to drive T_EM_ to T_RM_ conversion (66), the formation of blood-borne CD8^+^ T_RM_ cells in the lung likely depends on TNF, and its effect is prominent in previously infected lung tissues as compared to naive lung tissues (8). Because partner cells are also detected in the lung airways after parabiotic surgery (Figure 1) (6), circulating memory CD8^+^ T cells can reach to this tissue at basal levels, and CXCR3 plays a role in this recruitment (67). Treatment with pertussis toxin (PTx), which inhibits G protein-coupled chemokine receptors, significantly reduced the number of whole lung CD8^+^ T_RM_ cells (including the dynamic population in the airways), suggesting that not only migration from the lung interstitium to the airway, but also the entrance of circulating CD8^+^ T_EM_ cells into the lung depends on chemokine signaling (8). Despite their relatively low numbers, blood-borne lung CD8^+^ T_RM_ cells confer some extent of protection against respiratory virus challenge (61–63). It should be emphasized, however, that this protection is far inferior to that mediated by *bona fide* lung CD8^+^ T_RM_ cells generated by intranasal infection/immunization (5, 19, 23, 24, 48, 49). It is well known that the phenotype and function of memory CD8^+^ T cells in the circulation continues to change over time after infection, with central memory T cells (T_CM_ cells) emerging as the predominant subset (64, 68–70). This leads to reduced numbers of memory CD8^+^ T_EM_ that can be recruited to the lung and the eventual loss of a dynamic population of memory CD8^+^ T cells in the lung (8).

## Future Perspective

**Figure 2:**
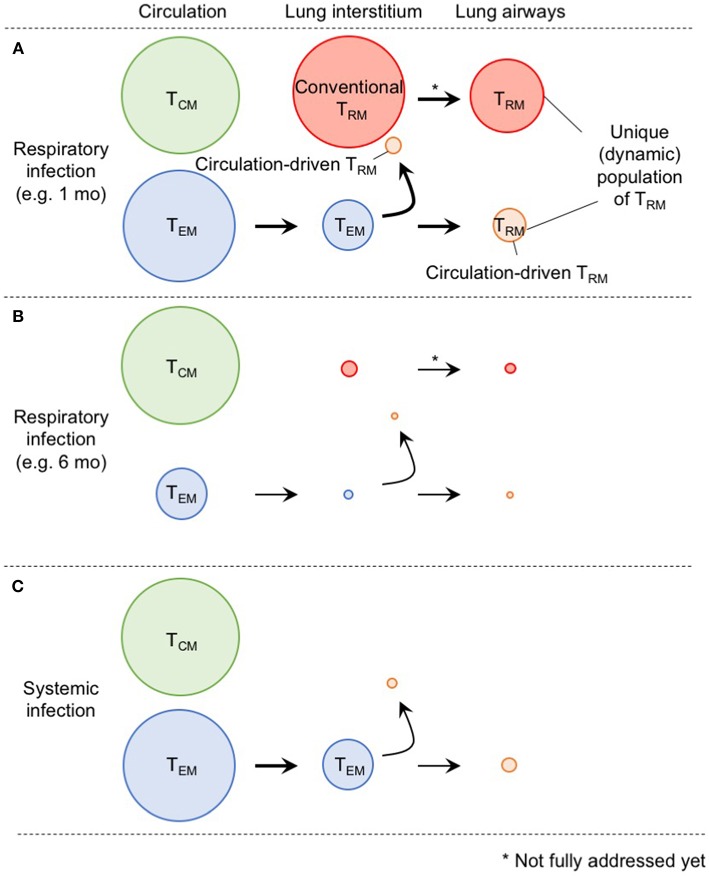
A comprehensive picture of memory CD8^+^ T cell populations in the lung. **(A)** Memory CD8^+^ T cells in the lung interstitium comprise a major population of conventional T_RM_ and a smaller population of T_EM_. Some of the latter also give rise to T_RM_ in response to TNF secreted in the conditioned lung that experience prior virus infection. Both host and partner cells in the interstitium are likely recruited to the lung airways and undergo phenotypic changes induced by environmental factors in this tissue. Although lung airway memory CD8^+^ T cells represent non-circulating population, and thus, are recognized as T_RM_, continual replacement is required for their maintenance. The size of the circles indicates the relative sizes of the respective populations in the lung. **(B)** As T_EM_ cells in the circulation decrease overtime after infection, input of T_EM_ to the lung interstitium and airways also decrease. Full recovery from the tissue damage, and resultant decrease of the size of RAMDs leads to reduction in the number of host CD8^+^ T_RM_ cells in the lung interstitium and airways. Consequently, the animals lost CD8^+^ T cell-mediated protective immunity in the lung. **(C)** Because of the lack of local antigen, *bona fide* CD8^+^ T_RM_ cells are not generated in the lung interstitium and airways. Although some T_EM_ cells give rise to T_RM_ in the lung, the extent is less than infection-experienced lung.

In Figure 2, we suggest a model by which the diverse populations of memory CD8^+^ T cells are generated and maintained in the distinct compartments of the lung. Although the ontogeny of lung T_RM_ and T_EM_ differs, some levels of conversion from T_EM_ to T_RM_ occurs within the lung interstitium and also following recruitment to the airways. Furthermore, although lung airway memory CD8^+^ T cells are a non-circulating population, the maintenance of their numbers depends on the continual influx of new cells from the lung interstitium. Thus, precise discrimination of each population is critical for future studies to avoid confusion in the field (2). Based on the model, it is likely that the limited longevity of conventional lung CD8^+^ T_RM_ cells and eventual loss of blood-borne lung CD8^+^ T_RM_ cells both contribute the rapid decay of total CD8^+^ T_RM_ cells in this tissue (Figure 2). In other words, such a short-lived nature of lung memory CD8^+^ T cells may, in a sense, be programed to avoid unnecessary pathogenesis in this tissue (71). Hence, multiple combinations of strategies to extend the longevity of both T_RM_ and T_EM_ should be considered for the development of vaccines against respiratory infectious pathogens. Since additional tissue damage is required to create new T_RM_ niches, strategies that enable the effective establishment of T_RM_ (including conversion from T_EM_ to T_RM_) without the induction of undesirable pathogenesis should be considered in the future.

## Ethics Statement

The studies utilizing laboratory animals were carried out in strict accordance with the Act on Welfare and Management of Animals of the government of Japan and the Regulations for the Care and Use of Laboratory Animals of Kinki University. The protocol for the present study was approved by the Institutional Animal Experimentation Committee of Kinki University Faculty of Medicine (Permit Number: KAME-26-025). All surgery was performed under anesthesia, and all efforts were made to minimize suffering.

## Author Contributions

All authors listed have made a substantial, direct and intellectual contribution to the work, and approved it for publication.

### Conflict of Interest Statement

The authors declare that the research was conducted in the absence of any commercial or financial relationships that could be construed as a potential conflict of interest.
